# Evaluation of the stiffness of normal cervix and its change with different factors using transvaginal two-dimensional shear wave elastography under strict quality control

**DOI:** 10.1186/s12880-023-01020-7

**Published:** 2023-05-22

**Authors:** Hui-Ping Zhang, Jing-Jing Wu, Wen-Ying Zhang, Jiu-Zhi Tao, Cheng-Bin Ma, Yu-Qing Zhou

**Affiliations:** 1 Department of Ultrasound, Shanghai Changning Maternity and Infant Health Hospital, East China Normal University, Shanghai, 200050 China; 2 Department of Gynecology, Shanghai Changning Maternity and Infant Health Hospital, East China Normal University, Shanghai, 200050 China

**Keywords:** Cervix, Two-dimension, Shear wave elastography, Transvaginal ultrasound, Quality control

## Abstract

**Background:**

The usefulness of transvaginal two-dimensional shear wave elastography (2D SWE) for cervical lesions is still uncertain. This study was to explore the value of transvaginal 2D SWE in the evaluation of the stiffness of normal cervix and its change with different factors under strict quality control (QC).

**Methods:**

Two hundred patients with normal cervix were included in this study and were examined using quantitative 2D SWE to evaluate cervical stiffness and its change with different factors under strict QC.

**Results:**

Intra-observer concordance of transvaginal 2D SWE parameters in midsagittal planes were acceptable with intraclass correlation coefficients higher than 0.5. Transvaginal 2D SWE parameters were significantly higher than the corresponding transabdominal parameters. 2D SWE parameters of internal cervical os were significantly higher than the corresponding parameters of external cervical os in a transvaginal midsagittal plane. 2D SWE parameters of external cervical os increased significantly over 50 years old, while these parameters of internal cervical os didn’t change significantly with increasing age. 2D SWE parameters of internal cervical os of horizontal position cervix were significantly higher than those of vertical position cervix. SWE parameters of normal cervix did not change according to different menstrual cycles, parities and human papilloma virus test results.

**Conclusions:**

Transvaginal 2D SWE under strict QC could provide quantitative, repeatable and reliable cervical stiffness information. Internal cervical os was stiffer than external cervical os. Menstrual cycles, parities and human papilloma virus test results wouldn’t affect cervical stiffness. However, age and cervical positions should be taken into condition while interpreting 2D SWE results of cervical stiffness.

## Background

Ultrasound elastography (UE), including strain elastography (SE) and shear wave elastography (SWE), could be used to evaluate the stiffness of living tissue. Compared with SE, which could provide qualitative or semi- quantitative elastic information, SWE, especially two-dimensional (2D) SWE, is a new elastic technique providing quantitative information of tissue stiffness [1, 2]. The application of SWE in liver, thyroid and some other organs has been with very promising results and its value has been widely accepted. Although it is undoubted that cervical lesions including cervical cancer could cause the changes of the stiffness, the value of SWE for cervical disease is still uncertain. There were only a few published studies exploring the value of SWE in the evaluation of cervical insufficiency and the prediction of the outcome of labor induction or the value of SWE in the diagnosis of cervical cancer [3–5]. And the guidelines and recommendations of World Federation for Ultrasound in Medicine and Biology and European Federation of Societies for Ultrasound in Medicine and Biology did not mention the possibility and value of SWE application in cervix [6, 7].

Unlike liver or thyroid, the stiffness of which is homogeneous; cervix is a complex and heterogeneous organ which is very important for women. The cellular portion of cervix is only 10%, consisting of smooth muscle cells mainly together with fibroblasts, epithelium and blood vessels; the rest 90% portion is extracellular matrix, consisting predominantly of collagen, especially type I collagen [8]. The ratio of extracellular matrix to smooth muscle cells is distributed differently at different parts of the cervix. The distal cervical portion has a greater ratio than the proximal portion [8]. And it is still uncertain if the cervical collagen contents and cellular contents may be changed or remodeled with age, parity, different menstrual cycle, pregnancy or delivery [9, 10].

To evaluate cervical stiffness with 2D SWE and to interpret 2D SWE results of cervical diseases, including preterm women, correctly, it is very important to understand 2D SWE results of normal cervix and how the factors including age, parity, phase of menstrual cycle and human papilloma virus (HPV) infection status affect the stiffness of normal cervix. Quality control (QC) for transvaginal 2D SWE is very important too, as some factors, such as the pressure exerted on the cervix through transducer and the movement of surrounding tissue could influence the accuracy of elastic results [11, 12].

So, in this study, we explored the value of 2D SWE in the evaluation of the stiffness of normal cervix and its change with age, parity, phase of menstrual cycle and HPV infection status under strict QC.

## Methods

### Study design

This was a cross-sectional observational study and was approved by the Ethics Committee of our hospital. Written informed consent was obtained from every patient before ultrasound examination.

### Patients

Outpatients in the Department of Gynecology at our hospital between January and February in 2021 were included in this study if they met the following criteria: (1) no contraindications for conventional transvaginal ultrasound (TVU) and no abnormal findings (including adnexal masses, endometrial lesions, myometrial lesions and cervical lesions) shown on conventional TVU; (2) without history of pelvic chemotherapy or pelvic radiotherapy; and (3) without history of any pelvic surgery except for cesarean section. The exclusion criteria were: (1) transvaginal 2D SWE not met the criteria of QC; (2) positive results for Thinprep cytologic test (TCT) or without TCT results; and (3) without HPV test results.

Age, menstrual cycle, parity and HPV test result of each patient were recorded.

The sample size was calculated according to the formula as N= (Z_a/2_σ/δ)^2^. In this formula, a = 0.05 (two tailed) and Z_a/2_ is 1.96; σ means the standard deviation (SD) for quantitative data, and here σ was 7 kpa according to our pre-experimental results; δ means absolute error, and here δ was 1kpa. According to the formula, the sample size should be 188 at least.

### Conventional ultrasound examinations

**Fig. 1:**
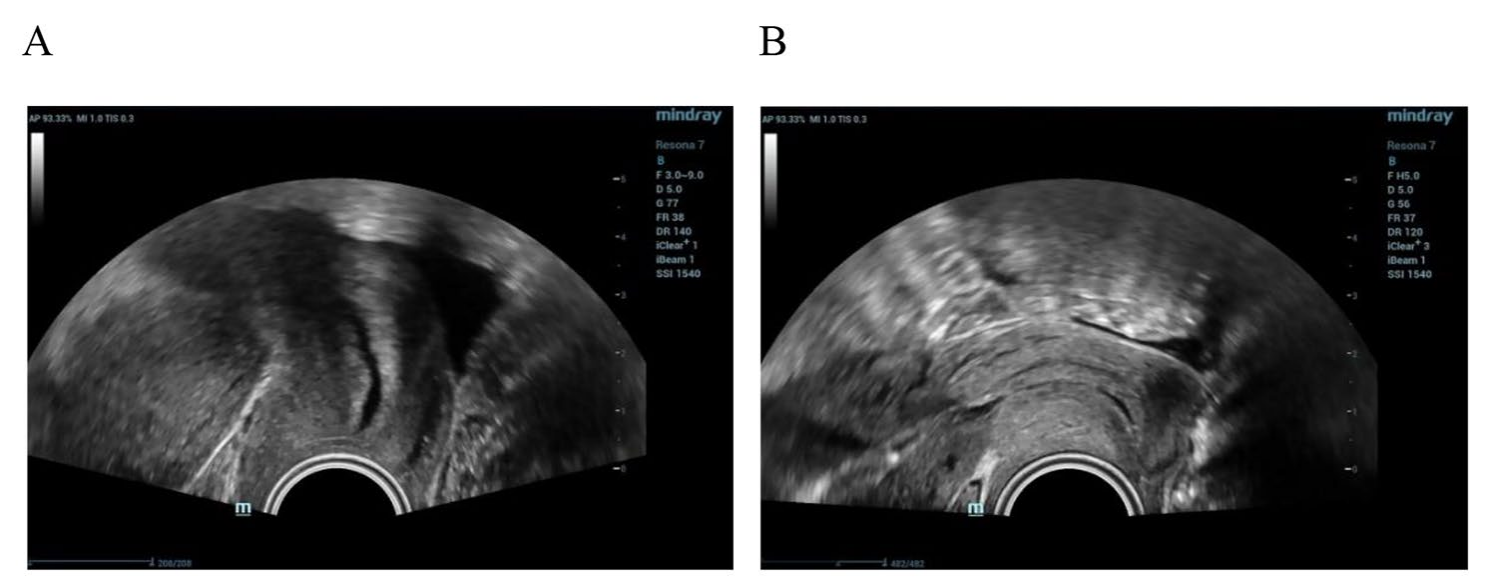
Different cervical positions. (**a**) A cervix with a vertical position. (**b**) A cervix with a horizontal position

All the ultrasound examinations were performed by a radiologist with 17 years’ experience in conventional TVU and 8 years’ experience in UE, including SWE. A Resona R9 diagnostic ultrasound system (Mindray Medical International, Shenzhen, China) with a transvaginal DE10-3WU probe and a transabdominal SC5-1U probe was used. The patient was instructed to empty the bladder before the ultrasound examination. A thorough conventional TVU scan was used first to exclude abnormal findings. Cervical position was observed and recorded as vertical position if the cervical canal was shown as comparatively vertical or as horizontal position if the cervical canal was shown as comparatively horizontal (Fig. 1).

### 2D SWE examinations

**Fig. 2:**
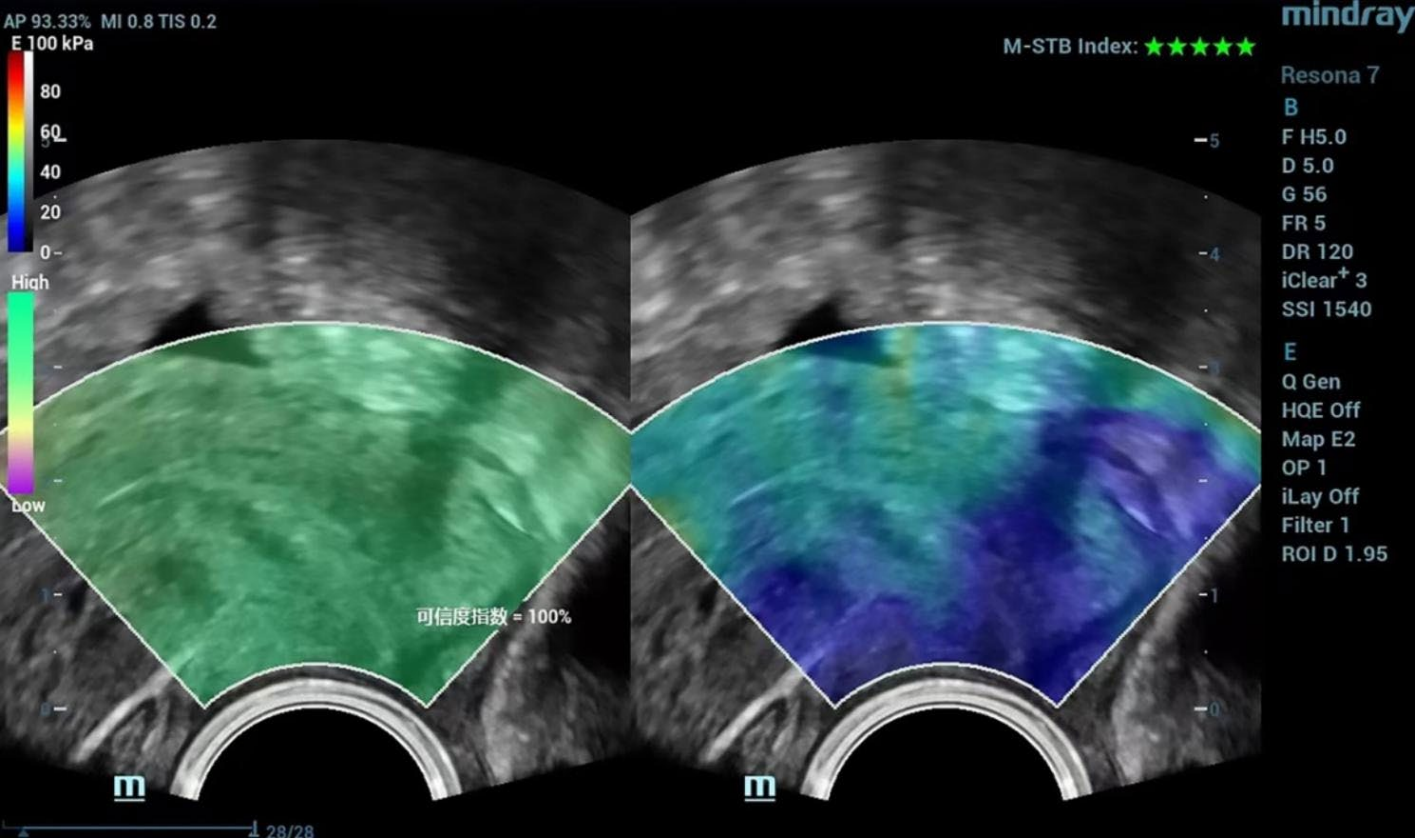
Two-dimensional shear wave elastography image under strict quality control with reliability index (RLB index) shown on the left as 100% and motion stability index (M-STB index) shown on the right as 5 green stars

**Fig. 3:**
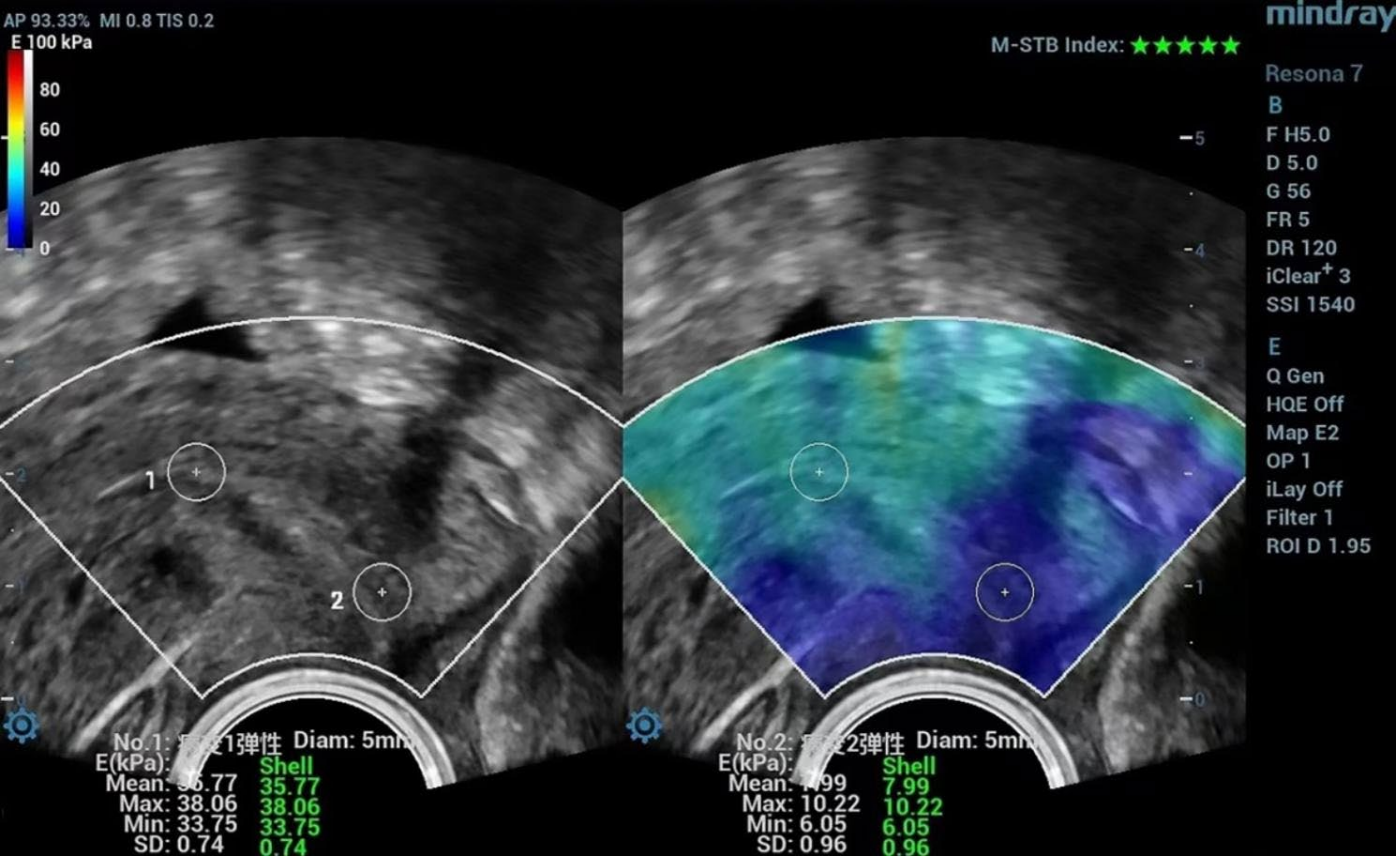
ROI drawing shown in the same figure in Fig. 2. Two circular ROIs with same diameter as 5 mm were put at internal and external cervical os respectively. Quantitative parameters as Emean (mean elasticity), Emax (maximal elasticity) and Emin (minimal elasticity) of each ROI were shown on the screen. ROI: region of interest

Before the study, the radiologist was specially trained in transvaginal 2D SWE examination. For transvaginal 2D SWE examinations, the transducer frequency was set as 5 MHz, the depth of the image as 5 cm and dynamic range as 120 dB. The probe was positioned in the anterior or lateral vaginal fornix gently with minimal pressure. A midsagittal plane of the uterine cervix was chosen first for SWE examination. The probe could be advanced or withdrawn to make sure the whole cervix including internal os and external os was shown on the screen. Region of interest (ROI) was set to include the whole cervix. The elasticity bar was set to a scale of 0-100 kpa with red color indicating hard tissue and blue color indicating soft. The ultrasound system we used provides two indices for QC as reliability index (RLB index, required to be higher than 95%) and motion-stability index (M-STB index, required to be 5 green stars, Fig. 2) [13]. For better image quality, the elastographic map should be hold stably for at least 3 s. When the image met the above quality, it was frozen and saved. Two circular ROIs with same diameter as 5 mm were put at internal and external cervical os respectively (Fig. 3). Quantitative parameters as Emean (mean elasticity in the ROI), Emax (maximal elasticity in the ROI) and Emin (minimal elasticity in the ROI) of each ROI were shown on the screen and recorded. Then, a transverse plane of external cervical os was chosen for SWE examination and a qualified image was frozen and saved. Two circular ROIs with the same diameter as 5 mm were put in the near field lip and far field lip of external cervical os respectively. Above mentioned quantitative parameters were acquired and recorded. Fifty patients were chosen randomly for the assessment of intra-observer consistency of cervical 2D SWE results.

A transabdominal scan was performed for each person. If the cervix could be displayed clearly at the depth less than 8 cm, a transabdominal 2D SWE examination was performed in a midsagittal plane of the uterine cervix. ROI was set to include the whole cervix. The elasticity bar was set to a scale of 0–30 kpa. If the image met above mentioned quality, it was frozen and saved. Two circular ROIs with the same diameter as 8 mm were put at internal and external cervical os respectively and above mentioned quantitative parameters were acquired and recorded. Transabdominal 2D SWE examinations were performed for fifty patients for the comparison between transabdominal and transvaginal results.

As Emean and Emax are the most commonly used SWE parameters and Emin is not widely used [6, 7], the outcomes of Emean and Emax were considered preferentially despite that we presented all the data of Emean, Emax and Emin.

### Statistical analysis

SPSS version13.0 software (IBM Corporation, Chicago, IL, United States) was used for statistical analysis. *P* < 0.05 was considered as statistically significant. Numerical variables were presented as mean ± standard deviation (SD), median (25th -75th percentile). Intraclass correlation coefficients (ICC) and their 95% confidence intervals (CI) were used for the analysis of the intra-observer consistency. Specifically, ICC with single measures and absolute agreement were used, applying two-way fixed effect models. Independent sample t test, paired t test and analysis of variance (with least significant difference post-hoc test) were used for the comparisons of numerical variables if they were versified with normal distribution (using Kolmogorov-Smirnov test); otherwise, nonparametric test including Mann-Whitney U test, Wilcoxon signed-rank test and Kruskal-Wallis H test (with Dunn post hoc tests) would be used instead.

## Results

### Basic information of the patients

There were 212 patients included in this study and transvaginal 2D SWE examination of cervix was performed for each patient. Five patients (5/212, 2.36%) were excluded as transvaginal 2D SWE could not meet the criteria of QC. Two patients were excluded for positive results of TCT test and five patients were excluded as they refused the TCT test.

So, there were 200 patients (aged 25–74 years; mean age 42.67 ± 12.32 years) in this study eventually. The patients were divided into three groups according to the age as ≤ 35 years group (n = 61), 36 ~ 49 years group (n = 73) and ≥ 50 years group (n = 66). Of the 200 patients, 78 patients were in proliferative phase, 52 were in secretory phase, 54 were in menopause and 16 were with irregular menstruation. Sixty-nine patients were nulliparous, 90 were primiparous and 41 were multiparous. Forty-two patients were HPV positive and 158 were HPV negative.

### Intra-observer consistency of transvaginal 2D SWE results

The intra-observer consistency of transvaginal 2D SWE quantitative results in 50 patients was shown in Table 1. The intra-observer concordances of the elastic quantitative parameters at the internal and external cervical os of midsagittal planes were acceptable (ICC > 0.50). For the transverse plane of external cervical os, the intra-observer concordances of the elastic quantitative parameters in the far field were acceptable (ICC > 0.50), too; however, the intra-observer concordances of the quantitative parameters in the near field were not good.

**Table 1:** The intra-observer concordance of transvaginal 2D SWE, shown as ICC (95% CI). N = 50

	Internal cervical os of midsagittal planes	External cervical os of midsagittal planes	Far field of external cervical os of transverse planes	Near field of external cervical os of transverse planes
**Emean**	0.733 (0.574–0.839)	0.587 (0.372–0.743)	0.728 (0.048–0.901)	0.216* (-0.066-0.465)
**Emax**	0.787 (0.654–0.873)	0.556 (0.331–0.721)	0.762 (0.485–0.881)	0.160* (-0.120-0.417)
**Emin**	0.667 (0.481–0.796)	0.611 (0.405–0.758)	0.555 (0.126–0.772)	0.329 (-0.058-0.554)

* means *P* > 0.05

2D SWE: two-dimensional shear wave elastography; ICC: intraclass correlation coefficients; CI: confidence intervals; Emean: mean elasticity; Emax: maximal elasticity; Emin: minimal elasticity

### Comparisons of 2D SWE outcomes between internal cervical os and external cervical os of transvaginal midsagittal planes

The 2D SWE quantitative parameters of internal cervical os and external cervical os of transvaginal midsagittal planes were shown in Table 2. The 2D SWE quantitative parameters of internal cervical os were significantly higher than the corresponding parameters of external cervical os.

**Table 2:** Comparisons of 2D SWE outcomes between internal cervical os and external cervical os of transvaginal midsagittal planes (kpa), shown as mean ± SD, median (25th -75th percentile). N = 200

	Emean*	Emax	Emin*
**Internal cervical os of** **midsagittal planes**	44.72 ± 6.40, 44.89 (41.50-49.59)	49.84 ± 6.29, 49.90 (46.17–54.01)	38.45 ± 7.26, 39.37 (34.91–43.53)
**External cervical os of** **midsagittal planes**	23.07 ± 9.24, 23.04 (15.15–28.91)	29.44 ± 9.35, 29.86 (22.07–35.99)	16.12 ± 8.71, 15.11 (8.92–21.81)
**t/Z**	12.259	30.188	12.252
***P***	< 0.001	< 0.001	< 0.001

* means non-normal distribution

2D SWE: two-dimensional shear wave elastography; SD: standard deviation; Emean: mean elasticity; Emax: maximal elasticity; Emin: minimal elasticity

### Comparisons of 2D SWE outcomes between transabdominal and transvaginal examinations

The transabdominal and transvaginal 2D SWE quantitative parameters in 50 patients were shown in Table 3. Transvaginal 2D SWE quantitative parameters were significantly higher than the corresponding transabdominal 2D parameters. And transabdominal 2D SWE quantitative parameters of internal cervical os had no significant difference with the corresponding parameters of external cervical os.

**Table 3:** Comparisons of 2D SWE outcomes between transabdominal and transvaginal examinations, shown as mean ± SD, median (25th -75th percentile). N = 50

	Internal cervical os of midsagittal planes			External cervical os of midsagittal planes		
	Emean*	Emax*	Emin*	Emean	Emax*	Emin*
**Transvaginal**	44.55 ± 7.04, 44.82 (41.68–48.76)	49.79 ± 7.06, 50.04 (47.01–53.97)	37.38 ± 7.45, 37.81 (24.17–41.41)	22.51 ± 10.11, 21.33 (14.94–29.47)	28.75 ± 9.62, 29.78 (21.01–35.74)	15.95 ± 8.74, 14.20 (10.26–21.39)
**Transabdominal**	8.54 ± 3.79, 7.91 (5.60-11.19)	11.25 ± 4.57, 10.34 (7.85–14.64)	5.86 ± 2.65, 5.12 (3.79–7.18)	8.06 ± 3.15^@^, 7.43 (5.45–10.11)	11.11 ± 4.50^@^, 10.80 (8.18–13.29)	5.63 ± 4.49^@^, 4.67 (3.11–6.64)
**t/Z**	6.154	6.154	6.154	11.004	6.115	5.565
***P***	< 0.001	< 0.001	< 0.001	< 0.001	< 0.001	< 0.001

* means non-normal distribution

^@^ means *P* > 0.05 compared with corresponding parameters of internal cervical os of transabdominal midsagittal planes

2D SWE: two-dimensional shear wave elastography; SD: standard deviation; Emean: mean elasticity; Emax: maximal elasticity; Emin: minimal elasticity

### Comparisons of 2D SWE outcomes among patients with different ages, cervical positions, parities, menstrual cycles or HPV infection states

The changes of 2D SWE parameters of transvaginal midsagittal planes with age, cervical position, parity, menstrual cycle and HPV infection were shown in Tables 4, 5, 6, 7 and 8.

**Table 4:** Comparisons of transvaginal 2D SWE outcomes among patients of different ages, shown as mean ± SD, median (25th -75th percentile)

Age	Internal cervical os of midsagittal planes			External cervical os of midsagittal planes		
	Emean	Emax	Emin*	Emean*	Emax	Emin*
**≤ 35 (n = 61)**	44.56 ± 6.80 45.11 (40.99–49.76)	49.64 ± 7.11 49.83 (44.62–54.38)	38.09 ± 7.38 39.27 (34.91–43.83)	20.37 ± 8.02^@^ 19.16 (14.18–26.75)	27.43 ± 8.88^@^ 26.85 (20.34–33.81)	12.65 ± 6.88^@^ 12.56 (7.78–16.89)
**35 ~ 49(n = 73)**	45.31 ± 6.97 47.25 (41.94–50.04)	51.14 ± 6.14 52.54 (47.93–54.60)	38.94 ± 8.13 40.28 (34.73–44.09)	22.27 ± 9.03 22.67 (14.90-29.33)	29.21 ± 8.78 30.41 (22.34–35.40)	15.54 ± 8.21^@^ 15.14 (9.05–21.55)
**≥ 50(n = 66)**	44.23 ± 5.31 43.36 (41.37–47.59)	48.60 ± 5.40 47.52 (44.67–52.65)	38.23 ± 6.11 38.91 (34.61–42.24)	26.45 ± 9.61 25.49 (20.65–33.74)	31.55 ± 10.06 31.30 (25.81–39.11)	19.98 ± 9.35 19.19 (13.66–25.83)
**F/χ** ^**2**^	0.518	2.942	1.123	12.603	3.177	21.431
***P***	0.596	0.055	0.570	0.002	0.044	< 0.001

* means non-normal distribution

^@^ means *P* < 0.05 compared with patients ≥ 50 years old

2D SWE: two-dimensional shear wave elastography; SD: standard deviation; Emean: mean elasticity; Emax: maximal elasticity; Emin: minimal elasticity

**Table 5:** Comparisons of transvaginal 2D SWE outcomes between patients with horizontal and vertical cervical positions, shown as mean ± SD, median (25th -75th percentile)

Cervical position	Internal cervical os of midsagittal planes			External cervical os of midsagittal planes		
	Emean	Emax	Emin*	Emean*	Emax	Emin*
**Horizontal position** **(n = 104)**	45.74 ± 7.30 46.26 (42.40-50.52)	51.33 ± 6.85 52.36 (47.29–55.13)	39.48 ± 7.98 40.17 (35.95–45.04)	21.88 ± 9.23 22.29 (14.49–27.77)	28.72 ± 9.41 29.37 (21.85–35.46)	14.76 ± 8.48 13.75 (8.00-20.56)
**Vertical position** **(n = 96)**	43.63 ± 5.07 43.51 (40.74–47.14)	48.23 ± 5.19 47.91 (44.67-52.00)	37.33 ± 6.23 38.00 (33.93–41.87)	24.36 ± 9.12 24.28 (16.49–32.08)	30.22 ± 9.27 30.02 (23.37–36.97)	17.60 ± 8.76 17.17 (10.65–23.36)
**t/Z**	2.393	3.633	2.563	1.792	1.131	2.289
***P***	0.018	< 0.001	0.010	0.073	0.259	0.022

* means non-normal distribution

2D SWE: two-dimensional shear wave elastography; SD: standard deviation; Emean: mean elasticity; Emax: maximal elasticity; Emin: minimal elasticity

**Table 6:** Comparisons of transvaginal 2D SWE outcomes among patients with different delivery times, shown as mean ± SD, median (25th -75th percentile)

Parity	Internal cervical os of midsagittal planes			External cervical os of midsagittal planes		
	Emean	Emax	Emin*	Emean*	Emax	Emin*
**0 (n = 69)**	44.40 ± 6.97 44.90 (40.43–49.60)	49.77 ± 6.95 49.94 (44.60-54.42)	38.33 ± 7.44 39.27 (35.24–43.84)	23.39 ± 9.12 23.49 (15.64–29.33)	30.42 ± 9.27 30.18 (23.30-37.46)	16.11 ± 8.94 14.77 (8.15–22.52)
**1(n = 90)**	44.97 ± 5.84 44.66 (42.15–49.17)	49.88 ± 5.85 49.57 (46.81–53.73)	38.69 ± 6.70 39.47 (35.33–42.69)	22.52 ± 8.59 22.85 (15.02–28.26)	28.30 ± 9.20 29.34 (21.30-33.97)	15.65 ± 7.92 15.03 (8.83–20.71)
**≥ 2(n = 41)**	44.74 ± 6.71 45.09 (39.62–50.01)	49.89 ± 6.20 50.58 (44.49–54.08)	38.10 ± 8.24 39.37 (32.83–44.32)	23.74 ± 10.27 23.96 (14.83–32.55)	30.30 ± 9.76 31.54 (22.35–37.27)	17.19 ± 10.03 16.24 (9.68–23.3)
**F/χ** ^**2**^	0.156	0.007	0.013	0.633	1.231	0.681
***P***	0.856	0.993	0.994	0.729	0.294	0.711

* means non-normal distribution

2D SWE: two-dimensional shear wave elastography; SD: standard deviation; Emean: mean elasticity; Emax: maximal elasticity; Emin: minimal elasticity

**Table 7:** Comparisons of transvaginal 2D SWE outcomes between patients in proliferative phase and secretory phase, shown as mean ± SD, median (25th -75th percentile)

Menstrual cycle	Internal cervical os of midsagittal planes			External cervical os of midsagittal planes		
	Emean	Emax	Emin*	Emean*	Emax	Emin*
**proliferative phase (n = 78)**	44.80 ± 6.79 45.25 (42.21–49.71)	50.12 ± 6.80 50.85 (46.80-54.49)	38.82 ± 7.73 40.13 (35.13–43.69)	21.23 ± 9.56 18.90 (14.38–27.02)	28.02 ± 9.18 27.80 (21.65–34.05)	14.61 ± 9.19 12.50 (7.73–19.55)
**secretory phase (n = 52)**	45.64 ± 6.97 46.22 (41.99–50.46)	50.92 ± 6.65 50.56 (47.04–55.44)	39.61 ± 7.20 41.22 (35.66–44.60)	23.23 ± 8.74 24.03 (15.24–29.61)	30.41 ± 9.51 31.75 (22.90-38.01)	15.55 ± 7.34 15.13 (9.32–21.48)
**t/Z**	0.678	0.661	0.891	1.404	1.431	1.290
***P***	0.499	0.510	0.373	0.160	0.155	0.197

* means non-normal distribution

2D SWE: two-dimensional shear wave elastography; SD: standard deviation; Emean: mean elasticity; Emax: maximal elasticity; Emin: minimal elasticity

**Table 8:** Comparisons of transvaginal 2D SWE outcomes between patients with and without HPV infection, shown as mean ± SD, median (25th -75th percentile)

HPV infection	Internal cervical os of midsagittal planes			External cervical os of midsagittal planes		
	**Emean**	**Emax**	Emin*	Emean*	**Emax**	Emin*
**+ (n = 42)**	45.20 ± 7.19 45.62 (41.16–49.90)	50.41 ± 6.39 49.22 (46.48–53.84)	39.04 ± 7.76 40.26 (35.01–44.13)	21.75 ± 10.26 21.01 (12.47–28.91)	28.46 ± 10.45 27.73 (18.85–36.88)	15.44 ± 10.05 12.31 (7.78–19.84)
**- (n = 158)**	44.60 ± 6.19 44.75 (41.53–49.17)	49.69 ± 6.27 50.02 (45.55–54.18)	38.29 ± 7.14 39.29 (34.83–42.69)	23.42 ± 8.94 23.97 (16.25–29.04)	29.70 ± 9.05 30.15 (22.42–35.66)	16.30 ± 8.35 16.23 (9.30-21.86)
**t/Z**	0.537	0.660	0.859	1.347	0.765	1.191
***P***	0.592	0.510	0.390	0.178	0.445	0.234

* means non-normal distribution

2D SWE: two-dimensional shear wave elastography; SD: standard deviation; Emean: mean elasticity; Emax: maximal elasticity; Emin: minimal elasticity

2D SWE parameters of external cervical increased significantly over 50 years old, while these parameters of internal cervical os didn’t change significantly with increasing age (Table 4).

2D SWE parameters of external cervical os (except Emin) for cervix with horizontal position and vertical position had no significant difference; however, 2D SWE parameters (Emean, Emax and Emin) of internal cervical os of horizontal position cervix were significantly higher than those of vertical position cervix (Table 5).

There were not significant differences of transvaginal 2D SWE quantitative parameters of both internal cervical os and external cervical os among patients with different delivery times (nulliparous, primiparous and multiparous) (Table 6). 2D SWE parameters of internal cervical os between patients in proliferative phase and in secretory phase had no significant difference, so did 2D SWE parameters of external cervical os (Table 7). And for patients with HPV positive or negative, 2D SWE parameters of internal cervical os had no significant difference, so did 2D SWE parameters of external cervical os (Table 8).

## Discussion

It is undoubted that cervical lesions including cervical cancer could cause the changes of the stiffness [14]. Transvaginal 2D SWE could provide quantitative information of cervical stiffness and would be helpful for the diagnosis and differential diagnosis of cervical diseases. It is essential for transvaginal 2D SWE examinations to be under strict QC and to grasp normal cervical stiffness results.

2D SWE software we used in this study was with strict QC including both credibility index and M-STB index shown on the screen. When images came up to the standard, the results we obtained were credible. Five patients (2.36%) in 212 were excluded from the study because they couldn’t meet the quality criteria. That is, the successful rate was as high as 97.64%. To improve image quality, the control of probe with minimal pressure to tissue and minimal movement is very essential; and the movement of surrounding intestine should be avoided. The patients were instructed to hold their breath during 2D SWE examinations if necessary. And the training of radiologists for transvaginal 2D SWE examinations was a critical factor, too [15].

Our results showed acceptable intra-observer consistency of quantitative 2D SWE parameters (Emean and Emax), ICCs higher than 0.7 at internal cervical os of midsagittal planes and ICCs higher than 0.5 at external cervical os of midsagittal planes. Intra-observer consistency of 2D SWE outcomes at external cervical os was not as good as that at internal cervical os. One probable reason was that external cervical os is usually too near to the probe, especially for cervix with a vertical position. The imbalance of ultrasound pressure in the near field may affect 2D SWE results and lead to the relatively worse intra-observer consistency. Furthermore, the intra-observer consistency of 2D SWE parameters of transverse planes was not as good as that of midsagittal planes, especially for the parameters in the near field. So, midsagittal planes would be the first choice for transvaginal 2D SWE examinations.

Transvaginal 2D SWE parameters of internal cervical os were significantly higher than the corresponding parameters of external cervical os in our study and this result was consistent with cervical physiology. Cervix is heterogeneous. Compared with proximal cervix, distal cervix has lower portion of smooth muscle cells. Our results were similar with the study of O’Hara S which showed that SWE parameters of internal os were significantly higher than those of external os though the ultrasound machine and SWE software they used were different with our study [16].

Transvaginal and transabdominal 2D SWE parameters were compared too. Transvaginal 2D SWE parameters were significantly higher than the corresponding transabdominal parameters though we used same sections under strict QC. Our results were partly similar with the study of O’Hara S et al. [17]. This result indicated that the elastic results from different probes could not be compared with each other even though the same ultrasound machine was used. And transabdominal 2D SWE quantitative parameters of internal cervical os had no significant difference with the corresponding parameters of external cervical os. This reason for that may be the long distance between the cervix and the probe as depth was an important factor that would influence SWE results [18, 19]. So, transvaginal 2D SWE would be a better choice for the evaluation of cervical stiffness, compared with transabdominal 2D SWE.

Our study also showed that women older than 50 were with higher external cervical stiffness than younger women while no significant differences were shown in internal stiffness with increasing age. This result was also consistent with cervical physiology as collagen content in cervix increases with age [10]. The study of Castro L et al. showed similar results as women older than 50 were with higher cervical stiffness than younger women [20]. However, the study of Thomas A et al. using semi-quantitative SE to evaluate normal cervical elasticity showed no significant changes with age [14]. This may imply that SE was not sensitive enough to reflect the changes of cervical elasticity with age. As cervical cancer mainly occurs at external os [21, 22], it is very important to understand the changes of external cervical os stiffness with age when using 2D SWE to diagnose cervical diseases.

Cervical positions would be taken into consideration when interpreting 2D SWE results according to our study. Though 2D SWE parameters of the external os for cervixes with horizontal position or vertical positions had no significant differences, Emean, Emax and Emin of the internal os for cervixes with horizontal position were significantly higher those corresponding parameters for cervixes with vertical position. One probable reason for this result would be the differences of ROI depths as internal os for cervixes with horizontal position would be nearer to the probe than internal os for cervixes with vertical position; as it is confirmed that ROI depths would affect the quantitative elastic results [23]. Another probable reason may be the effect of increased pressure putting on cervix as needed to acquire good image for vertical- position cervixes. The study of O’Hara S et al. described cervical positions as horizontal, angled and vertical, and their results showed that the internal os of a vertical cervix was more likely to be unsuccessfully examined using transvaginal SWE [16].

The cervical results of transvaginal 2D SWE of different menstrual cycles, different parities or HPV infection positive or not were compared in this study and our results showed no significant impacts of these factors on cervical 2D SWE results. The study of Castro L et al. observed increasing of cervical stiffness according to parity from nulliparity to multiparity [20]. Actually, the differences may be caused by the increasing of age as 73.9% (17/23) of nulliparous women were less than 35 year old and 58.8% (10/17) of multiparous women were more than 50 year old. Similar with our results, their study did not observe differences in cervical stiffness according to the menstrual phases and HPV infection state.

One limitation to our study was that the inter-observer consistency of transvaginal cervical 2D SWE was not evaluated. Considering the importance of QC and the influence that the operater may have on the results, it would be necessary to evaluate the inter-observer consistency in the next step.

## Conclusions

Transvaginal 2D SWE under strict QC could provide quantitative, repeatable and reliable cervical stiffness information. Internal cervical os was stiffer than external cervical os. Menstrual cycles, parities and HPV infection state wouldn’t affect cervical stiffness. However, age and cervical positions should be taken into condition while interpreting 2D SWE results of cervical stiffness.

## Data Availability

The datasets used during the current study are available from the corresponding author on reasonable request.

## List of abbreviations

UE: ultrasound elastography
SE: strain elastography
SWE: shear wave elastography
2D: two-dimensional
HPV: human papilloma virus
QC: quality control
TVU: transvaginal ultrasound
TCT: thinprep cytologic test
SD: standard deviation
ROI: region of interest
Emean: mean elasticity
Emax: maximal elasticity
Emin: minimal elasticity
CI: confidence intervals
ICC: intraclass correlation coefficients
